# Robust Stabilization and Synchronization of a Novel Chaotic System with Input Saturation Constraints

**DOI:** 10.3390/e23091110

**Published:** 2021-08-27

**Authors:** Ahmad Taher Azar, Fernando E. Serrano, Quanmin Zhu, Maamar Bettayeb, Giuseppe Fusco, Jing Na, Weicun Zhang, Nashwa Ahmad Kamal

**Affiliations:** 1 College of Computer and Information Sciences, Prince Sultan University, Riyadh 11586, Saudi Arabia; 2 Faculty of Computers and Artificial Intelligence, Benha University, Benha 13511, Egypt; 3 Instituto de Investigación en Energía, Universidad Nacional Autonoma de Honduras (UNAH), Tegucigalpa 11101, Honduras; serranofer@eclipso.eu or; 4 Research Collaborator, Prince Sultan University, Riyadh 11586, Saudi Arabia; 5 FET–Engineering, Design and Mathematics, University of the West of England, Bristol BS16 1QY, UK; Quan.Zhu@uwe.ac.uk; 6 Electrical Engineering Department, University of Sharjah, Sharjah 27272, United Arab Emirates; maamar@sharjah.ac.ae; 7 Department of Electrical and Information Engineering, Universita degli Studi di Cassino e del Lazio Meridionale, 03043 Cassino, Italy; fusco@unicas.it; 8 Faculty of Mechanical & Electrical Engineering, Kunming University of Science and Technology, No. 727 Jingming South Road, Chenggong, Kunming 650500, China; najing25@kust.edu.cn; 9 School of Automation and Electrical Engineering, University of Science and Technology Beijing, Beijing 100083, China; weicunzhang@ustb.edu.cn; 10 Faculty of Engineering, Cairo University, Giza 12613, Egypt; nashwa.ahmad.kamal@gmail.com

**Keywords:** chaos theory, bifurcation, stabilization, chaos synchronization, robust control

## Abstract

In this paper, the robust stabilization and synchronization of a novel chaotic system are presented. First, a novel chaotic system is presented in which this system is realized by implementing a sigmoidal function to generate the chaotic behavior of this analyzed system. A bifurcation analysis is provided in which by varying three parameters of this chaotic system, the respective bifurcations plots are generated and evinced to analyze and verify when this system is in the stability region or in a chaotic regimen. Then, a robust controller is designed to drive the system variables from the chaotic regimen to stability so that these variables reach the equilibrium point in finite time. The robust controller is obtained by selecting an appropriate robust control Lyapunov function to obtain the resulting control law. For synchronization purposes, the novel chaotic system designed in this study is used as a drive and response system, considering that the error variable is implemented in a robust control Lyapunov function to drive this error variable to zero in finite time. In the control law design for stabilization and synchronization purposes, an extra state is provided to ensure that the saturated input sector condition must be mathematically tractable. A numerical experiment and simulation results are evinced, along with the respective discussion and conclusion.

## 1. Introduction

Chaotic systems were studied several decades ago, as these types of systems are found in nature and other physical systems. Because of their wide range of applications, chaotic dynamical systems have received much attention over the last three decades. As the discovery of new physical systems in engineering and exact sciences is increasing, it is critical to suppress chaotic behavior because this phenomenon generates unwanted behavior when implementing this type of system. Furthermore, in recent years, the synchronization of chaotic systems has become critical in the synchronization of coupled chaotic systems, as found in applications such as like optics and other implementations in physics.

There are many results found in the literature related to different types of chaotic systems. This research study will mainly focus on self-excited attractors in their integer and fractional order. For example, the authors of [1] presented hidden and self-excited attractors for an oligopoly model by showing the respective bifurcation analysis. Another example is found in papers like that in [2], in which a describing function is implemented in a Chua circuit to find a hidden attractor. Then, in [3], a hidden chaotic attractor is found in a Lorenz system. In [4], the digital signal processing (DSP) implementation of fractional-order chaotic hidden and self-excited attractors is proposed.

Saturation is one of the constraints found in the input of many complex dynamic systems, something that can generate instability if a standard controller or synchronizer is implemented. There are a vast amount of research studies found in the literature which refer to this input nonlinearity. For example, in [5], a neural network control law is implemented for the stabilization and synchronization of a chaotic system with input saturation. Then, in [6], an adaptive controller is designed and presented for the control of a Lorenz chaotic system with input saturation. Other studies found in the literature are not necessarily related to the control and synchronization of chaotic systems where the design of appropriate controllers with input saturation is evinced. For example, in [7], the robust attitude control for a 3-DOF helicopter is shown when input saturation is presented in the system. Then, in [8], the saturation control of a switched system is given. In [9], the saturation control of a chain of integrators system is proposed. In [10], a robust controller for a class of nonlinear saturation system is studied.

Different control strategies for the stabilization and chaos suppression in this kind of system are essential considering that these control approaches provide a very efficient method when uncertainties or disturbances are found in a complex dynamic system [11]. The control of chaotic systems is crucial considering the physical applications in which this control strategy is needed. For example, in [12], a sliding mode controller and synchronization strategy for a chaotic system is designed based on a cubic reaching law. Then, in [13], a Duffing oscillator is controlled and stabilized by an active controller. Other examples are found in [14] in which an internal model principle does the robust control of a Chua circuit. Another example can be found in [15], where a robust controller is designed for a Lorenz system subjected to mismatched uncertainties. There are many control strategies found in the literature related to the control of many physical systems, such as in [16], in which a H-infinity controller is designed for a piezo electric actuator. Then, in [17], a robust controller for a permanent magnet synchronous motor is presented in which uncertainties are found in the system. Then, in [18], the suppression of chaos in a permanent magnet synchronous motor is achieved by a robust adaptive dynamic surface control.

The synchronization of a chaotic system is essential when two chaotic systems must be synchronized. Chaotic synchronization can be categorized as identical and non-identical [19,20,21,22,23,24,25]. There are plenty of papers in the literature in which this kind of synchronization controller strategy is evinced. Thus, for example, in [26], the inverse synchronization of two chaotic coupled systems with time delays are presented. Then in [27], the synchronization of a Chua system is achieved for a master–slave piecewise linear system. Finally, in [28], a Lorenz system is synchronized with an application to image encryption.

Other interesting results related to chaos stabilization are found in the litertaure shch as [29,30,31,32,33]. The studies like that in [34], in which a controller and stabilizer of magneto-elastic chaos found in a beam system, represented by the Duffing equation, is achieved by a delayed feedback control method. Then, in [35], the stabilization and synchronization of fractional-order discrete-time system is evinced. Another research study that reports interesting results is found in [36], in which a nonlinear dynamic inversion stabilizer and controller is obtained for a chaotic system. Then other results are found in studies like [37,38], in which in the first paper, period-doubling bifurcations are stabilized implementing smooth feedback, and in the second paper, a feedback controller to stabilize chaos synchronization is presented.

Moreover, there are significant research studies found in the literature related to chaos synchronization [39,40,41,42]. Therefore, for example, in [43], the synchronization and circuit design of an antimonotonic hyperjerk system is proposed in which reverse period-doubling bifurcation is used as a control parameter. Then, in [44], the synchronization of two Josephson junctions in series is presented. Another study related to synchronization is found in [45], where a complete synchronization is implemented for a system with non-inertial coupling. Finally, in [46,47], an output feedback controller for the chaos synchronization of stochastic reaction-diffusion time-delayed neural network and the chaos synchronization by optical feedback are presented, respectively.

In this paper, the stabilization and synchronization of a novel chaotic system are achieved by a robust controller. First, a novel chaotic system is presented to generate chaos by implementing a sigmoidal function. A dynamic analysis is done in which the equilibrium points, bifurcation analysis, and phase portraits are evinced to analyze the system’s chaotic behavior to show the domain of attraction, the system orbits, and the bifurcations of this novel chaotic system. The robust stabilization is done by selecting an appropriate robust control Lyapunov functional by adding an auxiliary system to find two control laws for chaos suppression purposes. The synchronization control law is obtained similarly by defining the system’s error variable. In this case, drive and response systems are defined, and then by selecting an appropriate robust control Lyapunov function and adding an additional system, two synchronization control laws are found. This research study presents two numerical experiments to validate the obtained theoretical results along with the respective discussion and conclusion. The self-excited attractor obtained in this research study provides a novel chaotic system that posses stability and chaotic region, as evinced in the bifurcation diagram, by estimating the parameters the chaotic regime is reached to generate an unstable equilibrium point.

One of the novelties of this new chaotic system is the use of a sigmoidal function which allows finding the chaotic regimen and behavior by approaching the equilibrium points as the domain of attraction. The sigmoid function nonlinearity can be switched such that the unstable limit cycle reaches the equilibrium points. It is important to mention that this novel’s chaotic system is probably extended to multi-wing, multi scroll, or even to a complex variable chaotic dynamic system, which could attract many researchers’ attention in chaos theory. Besides, the stabilization and synchronization in engineering-related physical systems can be achieved appropriately using several control techniques such as robust control, sliding mode control, and backstepping control. The control techniques for this kind of novel chaotic system can be implemented where the chaos is suppressed, or two identical systems are synchronized successfully even in the presence of nonlinearities.

As explained before, the contributions of this research study are that a novel chaotic system is provided for research purposes and modeling of many kinds of physical phenomena and other types of engineering-related systems, taking into consideration the dynamic behavior of this novel chaotic system. The input saturation in chaotic dynamic systems yields unwanted performance or even instability in this kind of chaotic system, so that a robust control is implemented for stabilization and synchronization of the novel chaotic system when saturation is found in the system inputs, so by a Lyapunov approach and by the use of the appropriate theoretical background, a robust and reliable control synchronization strategy is found.

The paper starts by evincing first the novel chaotic system. A sigmoidal function is used as a nonlinearity to drive the domain of attraction to the equilibrium points. Then, a bifurcation analysis reveals that this novel chaotic system becomes stable to chaotic by varying some system parameters, something essential to observe that a period-doubling occurs when the system enters a chaotic regime. Then, the robust stabilization of this novel chaotic system with input saturation is achieved by deriving a robust control Lyapunov function to find the appropriate control law that annihilates chaos and drives the system variables of this novel chaotic attractor their equilibrium points. A novel robust control synchronization law is found by obtaining the error of the system dynamic variables to obtain the synchronization law by designing the robust control Lyapunov function necessary to stabilize the error dynamics of the proposed novel chaotic system.

This research study aims to provide a novel fundamental theoretical background for chaos theory and its respective synchronization, anti-synchronization, and stabilization of any chaotic system. This work can be extended to other types of variations of chaotic systems, such as hyperchaotic systems, complex variable hyperchaotic systems, multi-wing, and multi-scroll chaotic system stabilization, synchronization, and anti-synchronization. The robust control strategy for synchronization and stabilization purposes provides a new theoretical framework useful for input saturated nonlinear dynamic systems. The selected robust Lyapunov function considers the system variables for stabilization purposes to reach the equilibrium points in finite time. The sector condition considers the saturated input to deal with this nonlinearity found in this chaotic system. An essential contribution of this research study is that synchronization of the two identical proposed systems is achieved by considering that the system input of the response system is saturated.

This paper is divided into the following sections. In Section 2, the related work to this study is presented, then in Section 3, the novel chaotic attractor is evinced. In Section 4, the main results of this study are shown. In Section 5, two numerical experiments are presented. Finally, in Section 6 and Section 7, the respective discussion and conclusion of this research study are presented.

## 2. Related Work

The dissipation properties of chaotic systems are one of the most important issues to consider in the dynamic analysis of chaotic systems. As shown in [48], the dissipation properties of a chaotic system’s convergence region or domain of attraction are contracted. As is well known, the divergence of a chaotic system’s vector field is given by [48]

(1) $$\nabla .F=\frac{\partial F}{\partial x_{1}}+\frac{\partial F}{\partial x_{2}}+\ldots +\frac{\partial F}{\partial x_{n}}$$

Meanwhile, for a chaotic system described by the following dynamic model $\frac{dX}{dt}=f\left(X,t\right)$, it is critical that this dynamic system meets the Lipschitz continuity property, which has been demonstrated in numerous papers in the literature, including in [49]:

(2) $$∥f\left(X_{s},t\right)-f\left(X_{m},t\right)∥\leq ∥L\left(X_{s}-X_{m}\right)∥$$

for a positive constant *L*. After considering the chaotic systems’ characteristics and properties, it is necessary to mention some research studies on chaos stabilization. As stated in the preceding section, it is critical to remember that the stabilization and control of various types of chaotic systems are critical, especially given that chaos suppression is one of the primary strategies that must be implemented to avoid this undesirable phenomenon. There are different research studies related to chaos stabilization, such as that in [50], in which a simple polynomial function of the system states is done by implementing adaptive feedback control for a chaotic system. Then, in [51], the control and anti-synchronization of a novel fractional-order chaotic system and its analysis are proposed. Another example is found in [52], where the chaos control of a fractional-order neural network with electromagnetic radiation is presented. Then, in [53], the chaos control of a piezoelectric auto parametric vibration system is shown.

The synchronization problem consists in following the drive system trajectory in time by the response system in order to drive the error variable to zero in finite time as appears in [28]:

(3) $$\epsilon =\sqrt{\sum_{i=1}^{n}{\left|Y_{Di}-Y_{Ri}\right|}^{2}}$$

where $Y_{D}\in R^{⋉}$ represents the discretized drive response in time and $Y_{R}\in R^{⋉}$ represents the discretized response system evolution in time. This root mean square error (RMSE) describes the accuracy of the control synchronizing strategy in order to make sure that the control synchronization will be the minimum and zero in finite time. Other results in the literature related to synchronization control of chaotic systems can be found in papers such as that in [54], which achieves chaos synchronization in the frequency domain. Then, in [55], a novel control approach does the synchronization of two non-identical 4-D hyperchaotic systems. Finally, in [56,57], the synchronization of two novel hyperchaotic system with unknown parameters and the circuit realization, control and synchronization of a novel hyperchaotic system are presented, respectively.

This study considers the discovery of a novel chaotic system, as well as the homoclinic and heteroclinic chaotic properties of this type of system. For example, in papers such as that in [58], it is demonstrated how some co-dimensions two bifurcations originate regions of chaotic and simple dynamics, and bifurcation structures such as Bykov T-points spirals are evinced. In papers such as that in [59], it is demonstrated how multiple chaos can arise from single parametric perturbations of a degenerated homoclinic orbit. The system under consideration in this paper is a periodical perturbed differential equation in which a Former’s perturbation is used to find the homoclinic orbits. Apart from the paper in [60], homoclinic chaos in piecewise smooth oscillators is demonstrated by using small parametric perturbations in this type of oscillator. In this paper, the Melnikov approach is used first to find that homoclinic chaos exists in a system without small perturbations, and then the same approach is used to find sufficient conditions to find the control parameters. Then, in [61], the homoclinic bifurcation and control of chaotic MEMS are presented. The Melnikov function is used as an analytical methodology for homoclinic chaos, which is expressed as an inequality in terms of system parameters. Another intriguing study can be found in [62], which shows novel bifurcation diagrams for piecewise smooth systems in which the transversability of homoclinic points does not imply chaos. Four scenarios are presented in this research study in which the systems are assumed to be subject to small non-autonomous perturbations, yielding four new bifurcation diagrams. Then, in [63], minimal topological chaos is discovered with respect to finite sets of homoclinic and periodic orbits.

Another important topic to mention is heteroclinic chaos, which is demonstrated in this paper through the design of a new chaotic attractor. There are numerous research studies in the literature that deserve to be mentioned in this research paper. In papers such as that in [64], for example, it is demonstrated that heteroclinic cycles connecting repellers and saddles in locally compact metric space induce chaos. The results presented in this paper are critical because they are based on a topological analysis in which the maps in the criteria are shown to have positive topological entropy and to be chaotic in the Li–Yorke sense. Then, in [65], another topological analysis is presented, in which chaos generated by heteroclinic curves connects repellers in complete metric spaces. This paper includes two classifications of heteroclinic cycles: regular and singular, as well as degenerated and nondegenerated. Other findings can be found in papers such as that in [66], which demonstrates a design methodology and algorithm for implementing geometric features with focus-saddle and center node equilibrium points.

Because entropy issues in novel chaotic systems are important in the theoretical development of this research study, it is worth mentioning some articles found in the literature on the entropy of novel chaotic systems. As an example, in papers such as that in [67], the enhancing of chaos complexity of a plasma model is demonstrated. It is demonstrated in this paper that by increasing the power input, the system can change from monostable to multistable without the addition of any input terms. It is also demonstrated that there is a transition, in terms of system complexity, from transient chaos to steady periodic behavior. Then, in [68], a novel measure inspired by Lyapunov exponents is demonstrated. In this paper, a network measure for characterizing state-transition networks is implemented. Another interesting study about chaotic system entropy found in the literature is that in [69], which shows evidence of strange attractors found in a C-Class amplifier with a bipolar transistor. The nonlinear bilateral behavior is shown in this paper to be a necessary but not sufficient condition for finding a complex behavior when the transistor is modeled as a two port admittance parameter. Meanwhile, the study in [70] demonstrates an important research study related to entropy in which the origin and fundamentals of entropy are presented, explaining how the entropy concept is used in physics, information theory, chaos theory, and data mining, and providing researchers with important issues that can be selected to choose the right variant of entropy for their research study. In addition, the study in [71] shows a chaotic time delay signature suppression by frequency band extracting is investigated the time delay signature and entropy grow enhancement in a chaotic optical feedback semiconductor laser. Then, in [72], a two-parameter bifurcation diagram for the computational analysis of Ca^2+^ oscillatory biosignals is presented. This paper explains how those types of diagrams provide different types of information about the analyzed autonomous system and how they complement one another. Aside from robust control, which is the strategy for stabilization and synchronization used in this research study, some research studies found in the literature related to the stability and chaos synchronization of various types of chaotic systems are worth mentioning. For example, in papers such as that in [73], the synchronization patterns in Kuramoto oscillators are demonstrated, in which phase locked states with constant phase shifts between these oscillators are studied. The synchronization estimation for complex time series using cross sample entropy measure is then demonstrated in papers such as that in [74]. Other papers with interesting results include that in [75], in which a modified Chua’s circuit is used with a five segment piecewise linear Chua’s diode. This paper demonstrates that the attractors have small basins of attraction. Finally, in [76], it is shown that a tuned pendulum absorber can reduce vibration and, at the same time to harvest energy. Then, in [77], it is shown how Lagrangian descriptors can be implemented to characterize invariant tori of generic systems. Finally, in [78], the magnetic confinement of a neutral atom is presented. In this paper, a neutral atom inside a double-wire waveguide in the presence of two uniform bias fields is presented.

It is important to remember that, in contrast to other research studies found in the literature, such as those in [72,73,74,76], the stabilization is done using techniques that do not guarantee stability and performance when saturation input is found in the respective chaotic systems shown in these papers. Note that although these control strategies do not take saturation into account, the high nonlinearity and complexity exhibited in these research papers make them ideal candidates for implementing robust control and synchronization strategies, but it is worth noting that there is sometimes a trade-off between the complexity of the designed controllers exhibited in these papers, so the authors consider. As previously stated, the obtained novel chaotic system shown in this paper can be used in engineering implementations as well as other exact science implementations such as metheorology, astrophysics, and astrochemistry.

Because of the high performance of the robust control synchronization law provided in this research study, the synchronization control law, as explained in previous sections, is achieved faster. Unfortunately, the literature on the implementation of robust control or other controller techniques such as sliding mode control and backstepping control for controller for chaotic system with input saturation is limited. Therefore, given the high nonlinearity of this novel chaotic system as a sigmoidal function, a roust control strategy is proposed to minimize the convergence error between the drive and response system variables until it reaches the origin and, as previously stated, faster than other types of control strategies that take into account the novel chaotic system’s high complexity.

Unfortunately, due to the lack of similar research studies found in the literature, a comparative analysis was not possible in the numerical experiment section, but as explained later in this paper, it is not necessary to do a comparative analysis taking into account that the stabilized and synchronized systems are novel chaotic systems, as observed in the numerical experiments.

## 3. Definition of the Novel Chaotic System

This section presents the definition of a novel chaotic attractor as well as a bifurcation analysis. The new chaotic attractor is made up of a sigmoidal function that causes the system to enter a chaotic state. The bifurcation analysis demonstrates how the system transitions to a chaotic regime and the period-doubling exhibited by the respective bifurcations. The applications of this novel chaotic system is very wide an can be implemented for the mathematical modeling of phenomenons found in meteorology [79], astrophysics [80], fluid dynamics [81], and in other engineering related systems such as electrical and power, mechanical, mechatronics, and chemical systems. The reason of the applicability of this novel chaotic system is that by estimating the right parameters and considering some topological aspects such as the limit set related to the unstable limit cycle and the domain of attraction this chaotic system can be implemented in the mathematical modeling of different types of phenomena.

### 3.1. Definition of the Novel Chaotic System

Consider the following novel chaotic attractor with sigmoidal functions:

(4) $$\begin{array}{ccc} {\dot{x}}_{1} & = & asigmoid\left(z_{1}\right)x_{1}-z_{1}^{3}\\ {\dot{y}}_{1} & = & bz_{1}+x_{1}y_{1}\\ {\dot{z}}_{1} & = & csigmoid\left(x_{1}\right)+dx_{1}y_{1} \end{array}$$

in which sigmoid is the sigmoidal function $sigmoid\left(x\right)=1/\left(1+e^{-x}\right)$. The equilibrium point of this system is ${\left[x_{1},y_{1},z_{1}\right]}^{T}={\left[-2.3,0.78,1.21\right]}^{T}$ for a system with initial conditions ${\left[x_{1}\left(0\right),y_{1}\left(0\right),z_{1}\left(0\right)\right]}^{T}={\left[-2.4,0.8,1.2\right]}^{T}$. The equilibrium points were obtained by the numerical nonlinear algebraic equation solver of GNU Octave *fsolve* of GNU Octave version 4.2.2. The set of nonlinear algebraic equations is done by considering the vector field $f(x_{1},y_{1},z_{1})=0$ so by solving this equation the equilibrium points are found efficiently. The values of the parameters used for this novel chaotic system are as follows: a=−1, b=4,9, c=13, and d=1 in order that the system reach the chaotic regimen using this parameters in all the numerical experiments of this paper. The Lyapunov exponents of this chaotic system are $L_{1}=-0.00195243$, $L_{2}=0.00852359$, and $L_{3}=-0.00394307$ verifying that only the Lyapunov exponent $L_{2}$ has a positive real component so the system is considered as chaotic. It is important to remark that by changing the parameters of this novel chaotic system the system solution can be varied from a quasiperiodic orbit to a chaotic orbit. The divergence of the system (4) is obtained in the following way:

(5) $$\nabla .F\left(x_{1},y_{1},z_{1}\right)=\frac{a}{1+e^{-z_{1}}}+x_{1}$$

The range of *a* in which the proposed novel chaotic system is dissipative is shown as follows:

(6) $$-max\left(x_{1}\left(1+e^{-z_{1}}\right)\right)<a<-min\left(x_{1}\left(1+e^{-z_{1}}\right)\right)$$

In this range of the constant *a*, the energy of the system dissipates until the equilibrium point reach the equilibrium points, so in this way, the periodic orbits of the novel chaotic attractor reach the equilibrium points. By this dissipation proof, it is verified and validated that the the dissipation characteristics for the novel chaotic system are found by establishing the range of the constant *a* in which this property is found.

**Figure 1:**
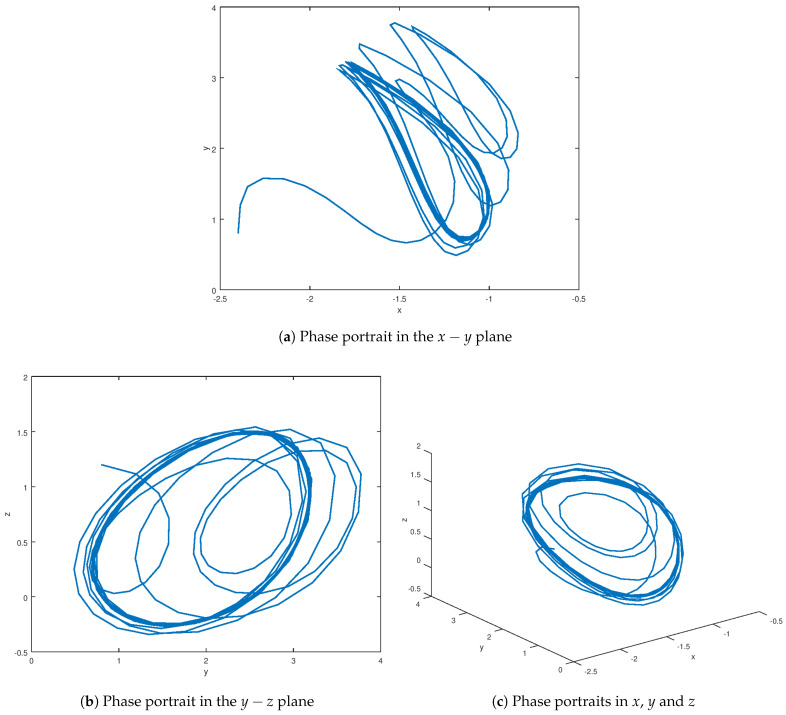
Phase portrait of the novel chaotic attractor (4).

The phase portraits in different planes are evinced in Figure 1 which corroborates the chaotic behavior of the novel chaotic system. It is possible to observe the orbit of this chaotic system, which follows a helicoidal trajectory around the domain of attraction, which is the equilibrium point. A vortex is formed around the domain of attraction, which is desirable in order to model certain natural systems. It is important to notice that the sigmoidal nonlinearities found in this novel chaotic systems yields the trajectory of the orbits of this proposed system to the equilibrium point in finite time. As verified later, in the bifurcation diagrams, the unstable limit cycle generated by chaos is obtained by selecting the appropriate parameters values in order to obtain the chaotic orbit. Due to the appropriate selection of the parameter values, the novel chaotic system enters in chaotic regimen considering that the initial conditions are crucial to find other domains of attractions in other equilibrium points. Because the domain of attraction in different equilibrium points of the novel chaotic systems is sensitive to the initial conditions, the initial conditions were chosen as long as they are close to the first encountered equilibrium point by determining that the inner product of two vector fields over the inner product of the initial condition and the equilibrium points is equal to infinity [82].

### 3.2. Bifurcation Analysis

**Figure 2:**
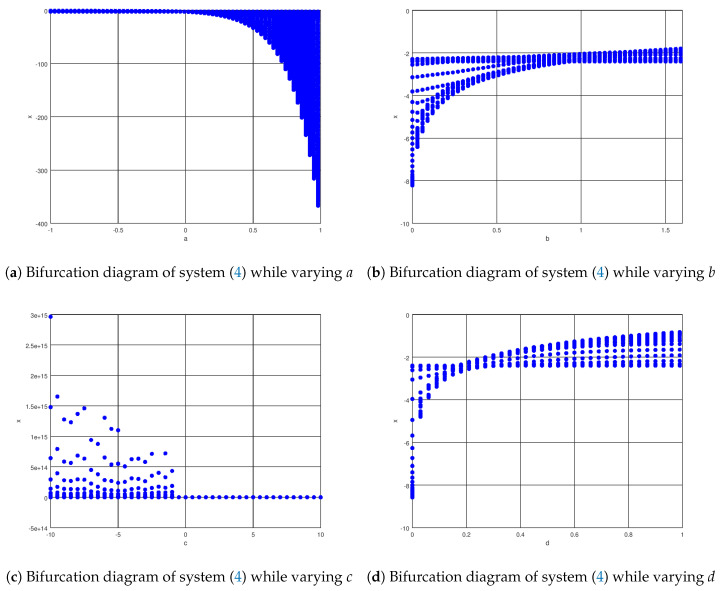
Bifurcation diagrams of the novel chaotic system (4).

The bifurcation diagrams while varying the constants *a*, *b*, and *d* in (4) are shown in Figure 2 in which it can be shown how the system states are driven from the stable region to the chaotic regimen by varying the parameters *a*, *b*, and *d*, as shown in the three figures. It is also demonstrated that, despite the fact that this is a novel chaotic system, the system exhibits chaotic behavior because period doubling occurs in the bifurcation diagram until the limit cycles appear in the novel chaotic system proposed in this paper.

The resulting diagrams demonstrate that the parameters *a*, *b*, and *d* can influence chaotic behavior; however, as explained later, there is one parameter that influences the system variables even more than the others in order to drive the system variables from the stable region to the chaotic regime. It is important to remark that while varying the parameter *a*, specifically when this parameter increases, the tendency of the variable *x* is to increase. This occurs because the parameter *a* is the only parameter that has to do with the divergence of this novel chaotic system as explained in (5) with the range (6), so the variable increases when it is outside the limits of the parameter *a*.

In Figure 2a, it can be seen how the bifurcation diagram is stable until it reaches the chaotic regimen in approximately a=0.3, and it can be seen that this parameter affects the transition between the stable region and the chaotic regimen the most. For the bifurcation diagrams shown in Figure 2b,d the bifurcations in chaotic regimen can be seen until stability is reached around b=1.3 and d=0.8. Meanwhile in Figure 2c it can be noticed the transition from the chaotic regimen to stability in the interval −10≤c≤0 while stability is found in the rest of the regions as noticed in the bifurcation diagram.

It can be seen in these bifurcation diagrams that the density of these diagrams evinces how the chaotic regimen is reached as well as the effects of sigmoidal nonlinearity. One of the most important conclusions is that the novel chaotic system generates chaos, which is significant when considering not only the phase portrait diagrams, but also the bifurcation diagrams when the parameters of the novel chaotic system are varied.

As a result of this section, it is possible to confirm the existence of a novel chaotic system. It is worth noting that the presented nonlinear dynamic system employs sigmoidal nonlinearity to generate chaos. This designed novel chaotic system is very important because new phenomena are discovered in nature every day, so it is critical to provide new chaotic systems in order to model novel complex physical systems discovered in exact sciences and engineering.

Another significant contribution of the novel chaotic systems is that it can be extended to other types of chaotic-related systems, such as hyperchaotic, multi-wing, or multi-scroll chaotic systems, in the latest kinds of chaotic system is important because the sigmoidal nonlinearity can extend this results to the kind of desired chaotic system by following an approach similar to that seen in the chaotic oscillator hidden attractor found in [82].

The design methodology of the robust control and synchronization laws that are used to synchronize this novel chaotic attractor is explained in the following section. It is critical to consider the saturation input nonlinearity as explained in this section in order to perform the stabilization and synchronization so as to provide a theoretical framework for applied sciences such as physics, chemistry, and biology, as well as engineering applications that require the stabilization, chaos suppression, and synchronization of chaotic systems.

This paper demonstrates that, regardless of the complexity of the input nonlinearity found in chaotic systems, it can be efficiently addressed by selecting the appropriate mathematical framework to stabilize and synchronize the proposed chaotic systems. Finally, it is validated how the designed controller and synchronizer stabilizes this novel chaotic system in the equilibrium points, as discovered and explained in this section, by providing the respective numerical experiment and simulation framework.

## 4. Main Results

The robust control laws for the stabilization and synchronization of the novel chaotic system presented in this study are deduced in this section. The control laws are deduced into two theorems, and the following drive (7) and response systems (8) are taken into account for stabilization and synchronization. In this section, it is demonstrated how the control strategy for stabilization purposes is designed while taking into account the saturation input nonlinearities, which can cause instability and deteriorated performance. When this occurs in a chaotic system, the consequences can be significant.

Note that the robust control strategy was chosen with the complexity of the sigmoidal function nonlinearity in mind, which is part of the novel chaotic system explained and demonstrated in this research study. The stabilization of this novel chaotic system is an important contribution because the findings can be applied to hyperchaotic, multi-wing, and multi-scroll chaotic systems. Note that in the synchronization case, only the identical case is considered for the purposes of this paper, and the results obtained and presented in this paper can later be extended to the non-identical case.

A robust control Lyapunov function is designed for the stabilization case in order to find the control law that stabilizes the system at the equilibrium points. Note that if the chaotic system presented in this paper needs to be stabilized in a different domain of attraction or at a different equilibrium point, a regulator is required to drive the system variables to the desired final value. Note that in the case of the synchronization of the novel two identical chaotic systems, these results can be extended to non-identical systems and other types of coupled chaotic systems.

(7) $$\begin{array}{ccc} {\dot{x}}_{1} & = & asigmoid\left(z_{1}\right)x_{1}-z_{1}^{3}\\ {\dot{y}}_{1} & = & bz_{1}+x_{1}y_{1}\\ {\dot{z}}_{1} & = & csigmoid\left(x_{1}\right)+dx_{1}y_{1} \end{array}$$

(8) $$\begin{array}{ccc} {\dot{x}}_{2} & = & asigmoid\left(z_{2}\right)x_{2}-z_{2}^{3}+\phi \left(u_{1}\right)+\alpha_{1}\\ {\dot{y}}_{2} & = & bz_{2}+x_{2}y_{2}+\phi \left(u_{2}\right)+\alpha_{2}\\ {\dot{z}}_{2} & = & csigmoid\left(x_{2}\right)+dx_{2}y_{2}+\phi \left(u_{3}\right)+\alpha_{3} \end{array}$$

in which the function ϕ(.) is the saturation nonlinearity. For the derivation of the controller and the stabilization and synchronization purposes, the following extra system is needed:

(9) $$\begin{array}{ccc} {\dot{\alpha }}_{1} & = & v_{1}\\ {\dot{\alpha }}_{2} & = & v_{2}\\ {\dot{\alpha }}_{3} & = & v_{3} \end{array}$$

so the following vectors are considered $X_{1}={\left[x_{1},y_{1},z_{1}\right]}^{T}$, $X_{2}={\left[x_{2},y_{2},z_{2}\right]}^{T}$, $U={\left[u_{1},u_{2},u_{3}\right]}^{T}$ and $\alpha ={\left[\alpha_{1},\alpha_{2},\alpha_{3}\right]}^{T}$. Rearranging (7) and (8) yields

(10) $$\begin{array}{ccc} {\dot{X}}_{1} & = & F\left(X_{1}\right)=\left[\begin{array}{c} asigmoid\left(z_{1}\right)x_{1}-z_{1}^{3}\\ bz_{1}+x_{1}y_{1}\\ csigmoid\left(x_{1}\right)+dx_{1}y_{1} \end{array}\right] \end{array}$$

(11) $$\begin{array}{ccc} {\dot{X}}_{2} & = & G_{1}\left(X_{2}\right)+\phi \left(U\right)+\alpha \\ {\dot{X}}_{2} & = & \underset{G_{1}\left(X_{2}\right)}{\underset{︸}{\left[\begin{array}{c} asigmoid\left(z_{2}\right)x_{2}-z_{2}^{3}\\ bz_{2}+x_{2}y_{2}\\ csigmoid\left(x_{2}\right)+dx_{2}y_{2} \end{array}\right]}}+\underset{\phi (U)}{\underset{︸}{\left[\begin{array}{c} \phi (u_{1})\\ \phi (u_{2})\\ \phi (u_{3}) \end{array}\right]}}+\underset{\alpha }{\underset{︸}{\left[\begin{array}{c} \alpha_{1}\\ \alpha_{2}\\ \alpha_{3} \end{array}\right]}} \end{array}$$

For the saturation nonlinearity, consider the following sector condition property:

**Property** **1.** *Considering a saturation nonlinearity ϕ(U) and the deadzone nonlinearity $D_{z}\left(U\right)$ with system input U, the following sector condition is met:*

(12) $$U^{T}\left[U-D_{z}\left(U\right)\right]\geq 0$$

*with:*

(13) $$D_{z}\left(U\right)=U-\phi \left(U\right)$$

*with these definitions the robust control laws for stabilization and synchronization purposes can be defined.*

In the two theorems of this paper, it does not matter the values of the saturation because the sector condition ensures that for any value of the saturation the stability of the stabilizer and synchronizer in ensured in the presence of saturation input, as explained in this paper. The saturation function used in the whole paper is given by

(14) $$\phi \left(x\right)=\left\{\begin{array}{ccc} -d_{1} & \mathrm{IF} & x<-l_{2}\\ x & \mathrm{IF} & -l_{1}\leq x<l_{2}\\ d_{2} & \mathrm{IF} & x\geq l_{2} \end{array}\right.$$

### 4.1. Robust Stabilization of the Novel Chaotic System

**Figure 3:**
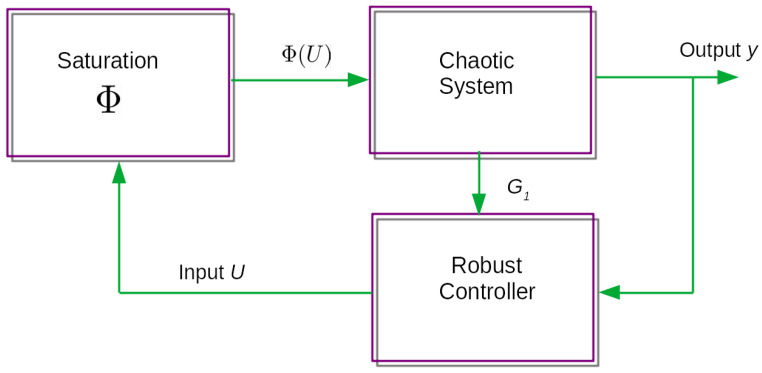
Block diagram of the closed loop stabilization controller.

The closed loop stabilization system’s block diagram is shown in Figure 3 where the robust controller and stabilizator are located in the feedback loop, connected to the saturation nonlinearity and receiving the vector field $G_{1}$. It is necessary to design the appropriate robust control Lyapunov function in order to obtain the respective control law that drives the system states to the equilibrium points in finite time in order to design the stabilization control law for this novel chaotic system. The robust control law obtained in this section includes a switching component, but no chattering is observed as a result of this. Consider the following theorem for the derivation of the robust control law for stabilization purposes.

**Theorem** **1.** *Consider the controlled system (11) and the extra system (9), so the following control laws stabilizes the novel chaotic system:*

(15) $$\begin{array}{ccc} U & = & -X_{2}\\ v & = & \frac{-\alpha }{{∥\alpha ∥}^{2}}X_{2}^{T}G_{1}\left(X_{2}\right)-X_{2} \end{array}$$

*By selecting an appropriate robust control Lyapunov function.*

**Proof.**  Consider the following robust control Lyapunov function:

(16) $$V=\frac{1}{2}X_{2}^{T}X_{2}+\frac{1}{2}\alpha^{T}\alpha$$

Now, by taking the time derivative of (16), the following results are obtained:

(17) $$\begin{array}{ccc} \dot{V} & = & X_{2}^{T}{\dot{X}}_{2}+\alpha^{T}\dot{\alpha }\\ \dot{V} & = & X_{2}^{T}G_{1}\left(X_{2}\right)+X_{2}^{T}\phi \left(U\right)+X_{2}^{T}\alpha +\alpha^{T}v\\ \dot{V} & = & X_{2}^{T}G_{1}\left(X_{2}\right)-U^{T}\left[U-D_{z}\left(U\right)\right]+X_{2}^{T}\alpha +\alpha^{T}v \end{array}$$

Then, by substituting the control laws (15) in (17) and by using the sector condition which appears in Property 1 the following result is obtained:

(18) $$\dot{V}=-U^{T}\left[U-D_{z}\left(U\right)\right]\leq 0$$

Therefore, with this conclusion the novel chaotic system is stabilized and the proof of the theorem is completed. □

As demonstrated in the previous theorem, it is possible to design a robust control law for the stabilization of the novel chaotic system.

### 4.2. Robust Synchronization of the Novel Chaotic System

**Figure 4:**
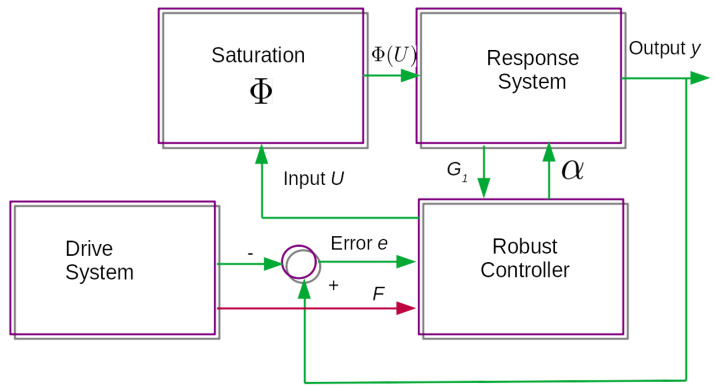
Block diagram of the control synchronization closed loop system.

The closed-loop synchronization controller for the novel chaotic drive and response system is shown in Figure 4. As can be seen, the system error, which is the difference between the state variables of the drive and response system, is used as input for the robust controller, and the additional state variable is also used for the synchronizer to drive the state error to zero in finite time. This input is applied to the input saturation function along with the input *U* to prevent saturation effects from causing instability or other undesirable effects. The robust control law for synchronization is then obtained by selecting an appropriate Lyapunov robust control law and then obtaining the first derivative of this Lyapunov function to obtain the synchronization control law. When the saturation nonlinearity is found in the response system input, the sector condition, as shown in Property 1 is critical in order to find the required stability conditions. As stated in the introduction section, the theoretical results presented in this paper are based on the identical chaotic system synchronization of the novel chaotic system described in Section 3. The most important results are evinced in the following theorem in this subsection, in order to provide the theoretical framework for these types of systems. Consider the following theorem for the Robust synchronization of this identical novel chaotic system.

**Theorem** **2.** *Considering the following error variable and its derivative:*

(19) $$\begin{array}{ccc} e & = & X_{2}-X_{1}\\ \dot{e} & = & G_{1}\left(X_{2}\right)+\phi \left(U\right)+\alpha -F\left(X_{1}\right) \end{array}$$

*so the following robust synchronization laws are obtained for systems (10) and (11):*

(20) $$\begin{array}{ccc} U & = & -e\\ v & = & \frac{-\alpha }{{∥\alpha ∥}^{2}}e^{T}\left[G_{1}\left(X_{2}\right)-F\left(X_{1}\right)\right]-e \end{array}$$

*By selecting an appropriate robust control Lyapunov function.*

**Proof.**  Consider the following robust control Lyapunov function:

(21) $$V=\frac{1}{2}e^{T}e+\frac{1}{2}\alpha^{T}\alpha$$

Now, by taking the time derivative of ( 21) yields

(22) $$\begin{array}{ccc} \dot{V} & = & e^{T}\dot{e}+\alpha^{T}\dot{\alpha }\\ \dot{V} & = & e^{T}\left[G_{1}\left(X_{2}\right)-F\left(X_{1}\right)\right]+e^{T}\left[U-D_{z}\left(U\right)\right]+e^{T}\alpha +\alpha^{T}v \end{array}$$

so by substituting (20) into (22) and by using Property 1 the following result is obtained:

(23) $$\dot{V}=-U^{T}\left[U-D_{z}\left(U\right)\right]\leq 0$$

so the robust synchronization stability is achieved and the proof is completed. □

This proof demonstrates how the closed-loop stability of the error variable dynamics is ensured in order for the state variables of the response system to reach the state variables of the drive system. A very accurate and fast robust control synchronization is achieved with the obtained control law and by the implementation of an extra variable that aids in the closed loop stability. As a result, it is important to remember that, similar to the stabilization case, the error system dynamics reach zero in finite time faster despite the presence of an input saturation nonlinearity in the system. Before concluding this section, note that the theoretical proofs evinced in two theorems allow for faster and more accurate stability of the closed-loop system in the stabilization and synchronization case. In the following section, two numerical experiments demonstrate how both of these control strategies, for stabilization and synchronization purposes, provide accurate results as demonstrated theoretically in this section.

## 5. Numerical Experiments

Two numerical experiments are presented in this section: the first for the stabilization of the novel chaotic system and the second for the control synchronization of two identical novel controllers. The novel chaotic system is robustly stabilized in the first experiment by selecting specific initial conditions with saturation input nonlinearity. In this manner, the robust controller used for stabilization is validated. In the case of robust synchronization, two novel chaotic systems are synchronized by a robust control law so that the response system variables follow the evolution of the drive system variables in time. This goal must be met despite the fact that the response system’s input is saturated nonlinear. In these numerical experiments, it is verified and validated that the robust control synchronization law successfully synchronizes both systems in order to reduce the synchronization error to zero.

### 5.1. Experiment 1: Robust Stabilization of the Novel Chaotic System

This experiment involves the robust stabilization of a novel chaotic system. Because the saturation nonlinearity is in the system’s input, the robust controller is used to overcome or outperform the performance of the closed loop system in the presence of this nonlinearity. This numerical example effectively validates the proposed control strategy. The response of the novel chaotic system variable is stabilized while eliminating or suppressing the chaotic behavior in this novel chaotic system, allowing the state variables to be driven to the equilibrium points in finite time without exhibiting chattering or other undesirable phenomena in this type of robust controller.

Note that if the initial conditions are close to another domain of attraction, the system variables will be stabilized in different equilibrium points. As shown in the block diagram in Figure 3, the vector field $G_{1}$ must be calculated in order to obtain the required control input action, implying that some kind of compensation is required to deal with the saturation input, as shown in the theoretical results obtained in this research study. The results of this numerical experiment are as follows: first, the evolution of the state variables over time, then the evolution of the input variables over time, and finally the respective phase portraits.

For the robust stabilization of the novel chaotic controller with input saturation (8), the following initial condition is considered $X_{2}={\left[-2.4,0.8,1.2\right]}^{T}$ with the saturation function:

(24) $$\phi \left(x\right)=\left\{\begin{array}{ccc} -10 & \mathrm{IF} & x<-15\\ x & \mathrm{IF} & -15\leq x<15\\ 10 & \mathrm{IF} & x\geq 15 \end{array}\right.$$

This saturation nonlinearity is found in the system input, as shown in Figure 3. It can be demonstrated that the theoretical derivation of the stabilization controller for this novel chaotic system ensures the overall system’s stability, given that due to a rigorous theoretical derivation, independent of the saturation parameters, the stabilization of the variables of this novel chaotic system is achieved in order for these state variables to reach the eigenvalues. The robust control law suppresses the instability by assuming that the overall closed-loop system is stabilized by an efficient Lyapunov design. In this numerical experiment section, it is demonstrated that the saturation nonlinearity found in the input of this novel chaotic system has no effect on the overall performance of the closed loop chaotic system, regardless of the saturation parameters. Because of an efficient controller design, the robust controller effectively overcomes saturation nonlinearity in order to stabilize the novel chaotic system in the equilibrium points. Note that in this numerical experiment, the stabilization occurs quickly and precisely, with no oscillations or chattering.

**Figure 5:**
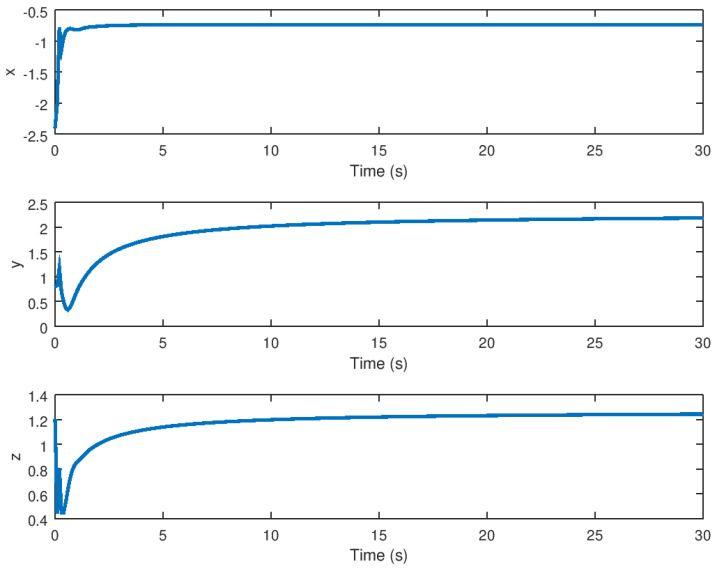
Evolution in time of the stabilized state variables of the novel chaotic attractor.

**Figure 6:**
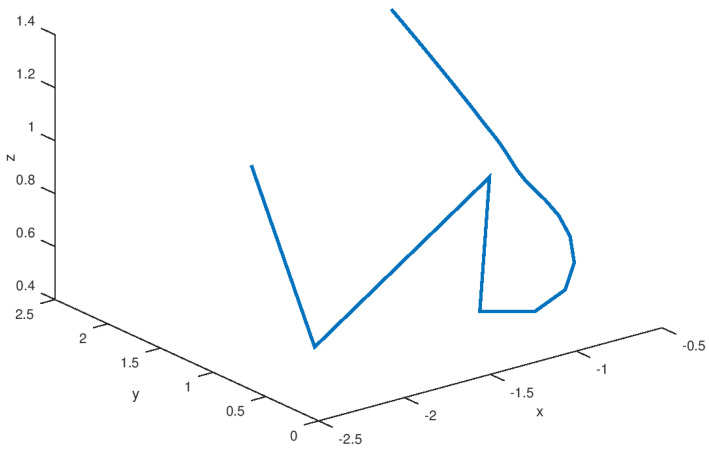
Stabilized phase portrait of the novel chaotic system.

The stabilized state variables *x*, *y*, and *z* are shown in Figure 5, and it is noted that these variables reach equilibrium in finite time without the presence of unwanted oscillation. It has been demonstrated that the robust stabilization controller completely suppresses the chaos behavior, demonstrating that the main purpose of this proposed controller has been met. As previously stated, the state variable evolution reaches the equilibrium points; however, by varying the initial conditions close to other domains of attraction, other equilibrium points can be reached using the same robust control law; this is due to the robust control law’s robustness. The Lyapunov functional design is independent of the equilibrium points, because the controller is designed in such a way that the domain of attraction related to the equilibrium point is close to the robust controller performance. The stabilized phase portrait is shown in Figure 6, and as can be seen, chaos suppression is used to eliminate all unwanted oscillations. The limit cycle vanishes when equilibrium is reached. In comparison to the phase portrait shown in Figure 1, it is demonstrated that this variable is stabilized along a smooth trajectory rather than the chaotic trajectory shown in the previous figure. As a result, the robust controller action eliminates the chaotic behavior satisfactorily, and the system variables shown in this phase portrait are efficiently driven to the equilibrium point.

**Figure 7:**
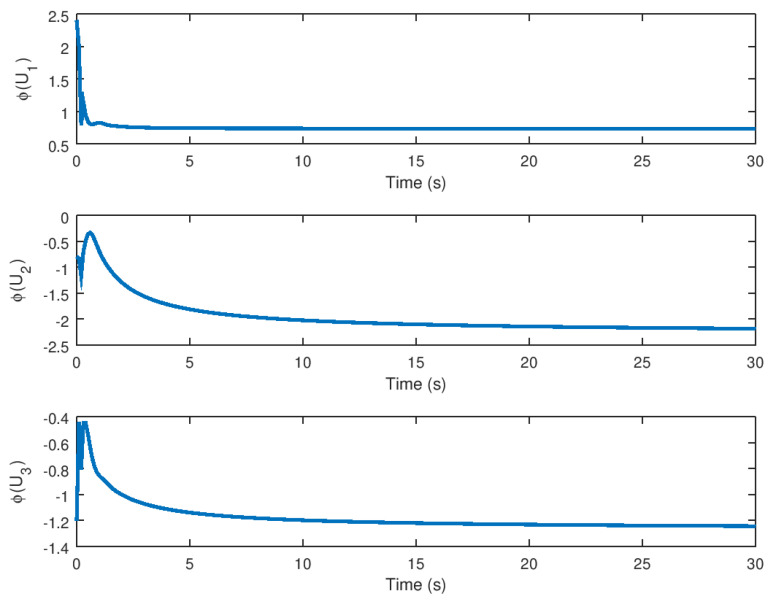
Input variable *U* of the novel chaotic system by the implementation of the proposed robust controller.

**Figure 8:**
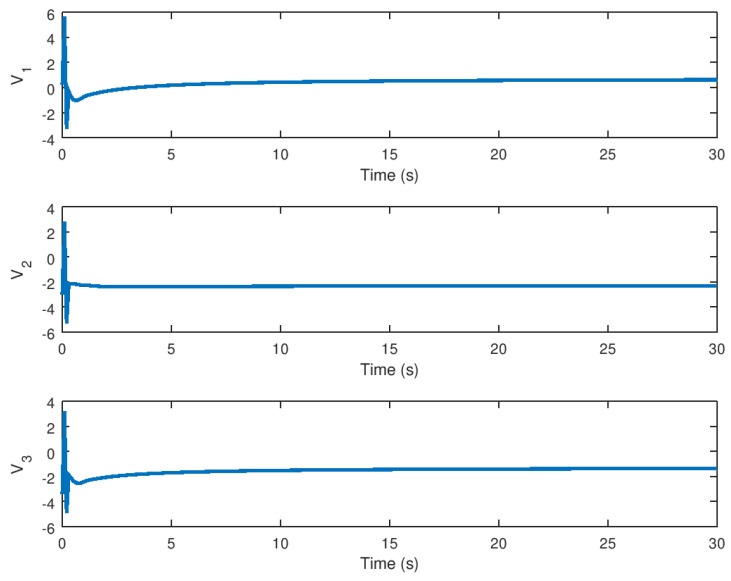
Input variable *v* of the novel chaotic system with the proposed robust controller.

The input variables *U* and *v* are depicted in Figure 7 and Figure 8, respectively. It is possible to see how these variables arrive at a final value until the trajectory or orbit of the novel chaotic system depicted in this paper reaches equilibrium. Note that these input variables provide a low control effort and chattering ausence, which demonstrates the high performance of the proposed robust controller for the stabilization of the novel chaotic system. Another important consideration is that the controller action would be significantly small control effort in the case that this robust control strategy is implemented in a real physical system, given that the saturation input nonlinearity yields instability and poor performance that can avoid chaos suppression in an efficient manner.

**Figure 9:**
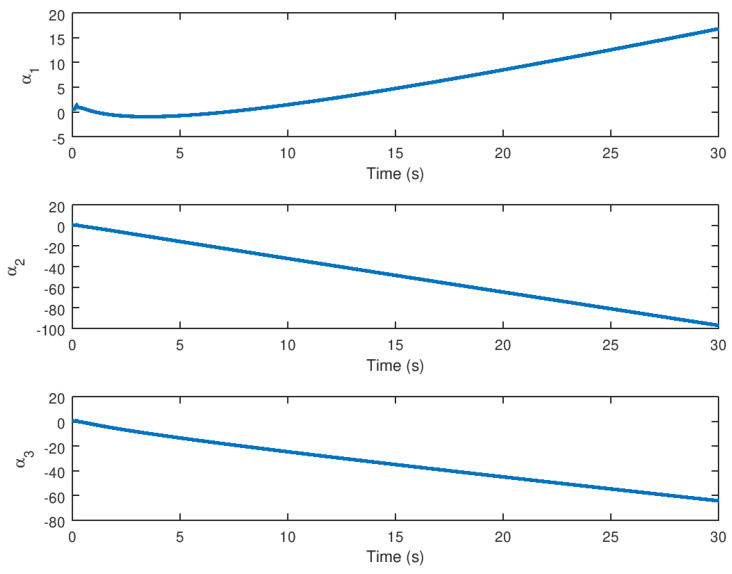
Evolution in time of the variable α for stabilization purposes.

Finally, as shown in Figure 9, the evolution in time of the auxiliary variable α for stabilization purposes is evinced in which is the extra state variable with input *v* as shown in Figure 8. When saturation is detected in this novel chaotic system, this provides adequate input for stabilization.

### 5.2. Experiment 2: Robust Synchronization of the Novel Chaotic System

The synchronization of two identical chaotic systems is demonstrated in this numerical experiment. This experiment’s chaotic system is a novel chaotic system shown in this paper. One system serves as a drive system, while the other serves as a response system. The saturation nonlinearity is found in the input of the response, as explained in the block diagram shown in Figure 4, so it is important to note that this kind of nonlinearity in the input of the novel chaotic system is not commonly found in the literature, but in real physical systems this phenomenon is commonly found considering the type of application. It is also worth noting that the vector fields *F* and $G_{1}$ must be calculated because, similar to the stabilization case, they act as a compensator to overcome the saturation nonlinearity regardless of its characteristics. The following initial conditions are implemented in this numerical experiment for the synchronization of two identical novel chaotic systems, where (10) is the drive system and (11) is the response system: $X_{1}={\left[-2.4,0.8,1.2\right]}^{T}$ and $X_{2}={\left[-2.9,0.4,1.7\right]}^{T}$ with the saturation input shown in (24).

**Figure 10:**
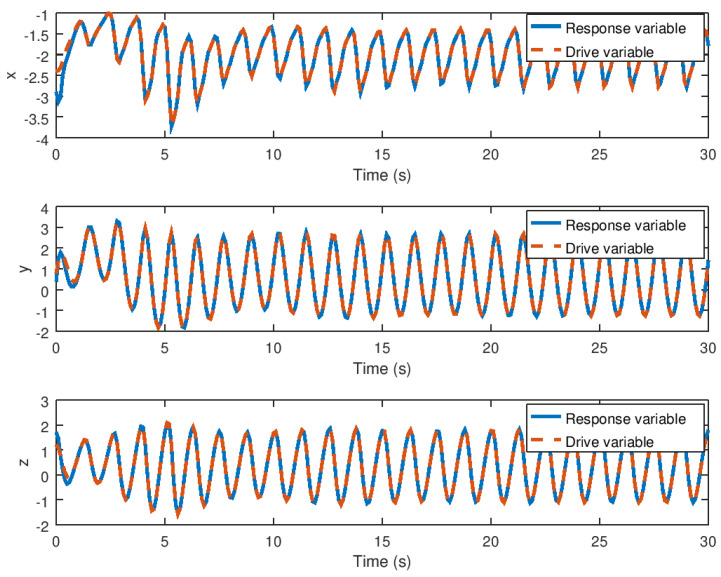
Evolution in time of the synchronized state variables of the drive and response novel chaotic system.

**Figure 11:**
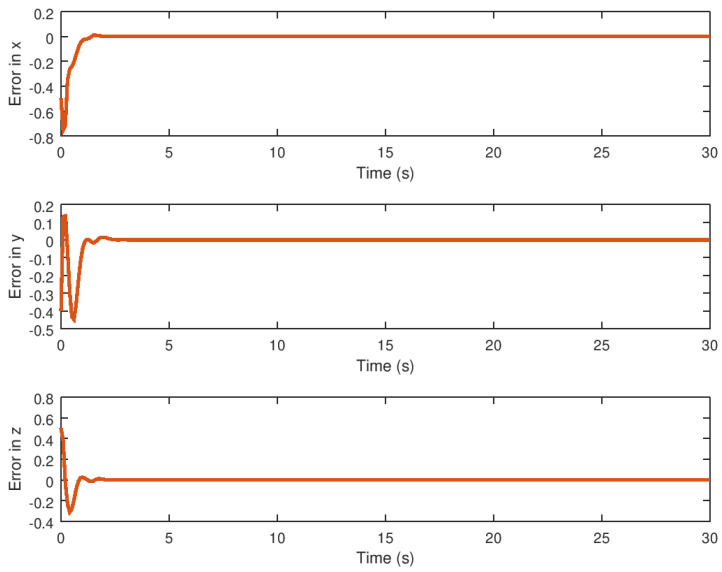
Evolution in time of the synchronization error variables.

The evolution in time of the synchronized state variables, as well as their respective error variables, is depicted in Figure 10 and Figure 11. As can be seen, the response variables *x*, *y*, and *z* reach the drive variable’s trajectory in finite time, whereas the error reaches the origin or zero value faster. Note in these figures that the robust controller performs optimally by rapidly and precisely driving the error variables to zero. Note that the proposed robust control synchronization strategy provides a fast and accurate system response even in the presence of saturation nonlinearity, regardless of the characteristics of this phenomenon found in the drive system’s input. It should be noted that this robust controller is only suitable for the synchronization of identical chaotic systems; for the synchronization of non-identical chaotic systems, this synchronization robust control strategy must be modified in order to achieve the required closed loop system performance.

**Figure 12:**
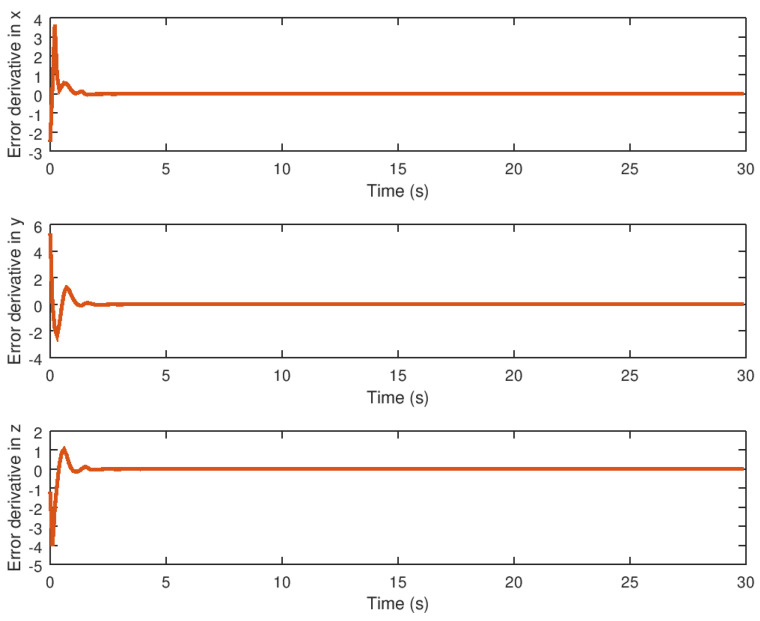
Derivative of the synchronization error variables.

The derivative of the error variable is shown in Figure 12. The velocity of error convergence is shown in this figure, indicating that the error variable reaches the origin in finite time faster due to the action of the robust control strategy even in the presence of saturation input nonlinearity. The peaks of the error variable derivative are thought to be small, which is related to the acceleration with which the error dynamics reach the origin in finite time, which is desirable when the proposed robust control synchronization strategy is implemented in real physical systems.

**Figure 13:**
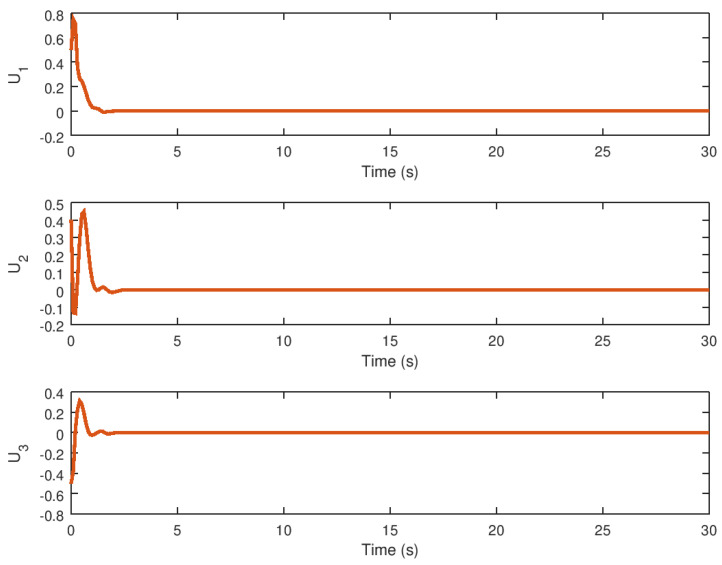
Input variable *U* for the response novel chaotic system.

**Figure 14:**
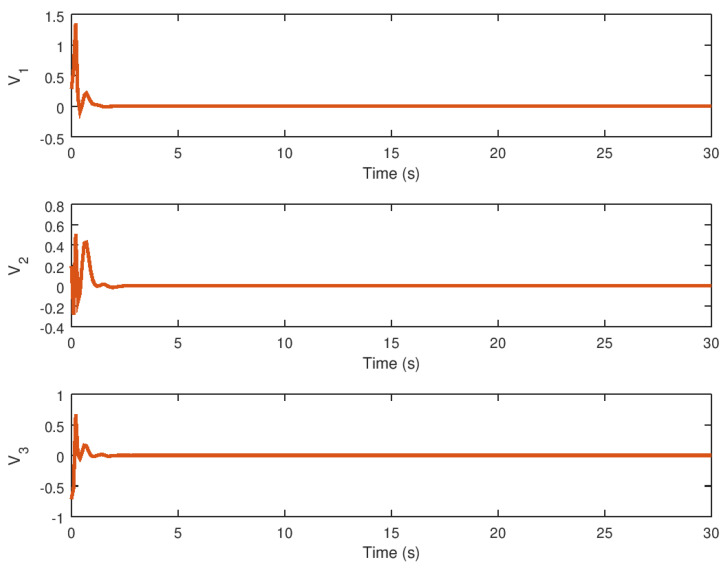
Input variable *v* for the novel chaotic response system.

The evolution of the synchronization input variables *U* and *v* in time is depicted in Figure 13 and Figure 14. As with the stabilization case, these input variables reach their final values in finite time until the drive and response variables are synchronized. Note that, despite the discontinuity in the robust controller part, the proposed control synchronization law does not produce chattering.

In these figures, the control variable *U* provides a non-oscillatory control action and a low control effort, which is important when this synchronization strategy is used in a variety of physical systems such as those found in physics, chemistry, biology, and engineering. Meanwhile, the time evolution of the virtual input variable *v* is shown, demonstrating that this error variable contributes only a minor control effort, implying that the overall control action must be as small as possible even in the presence of saturation input nonlinearity. Note that the control law design can be implemented in specific cases where the novel chaotic system must be synchronized in a real physical system or implemented in the form of a circuit or mechanical chaotic system.

**Figure 15:**
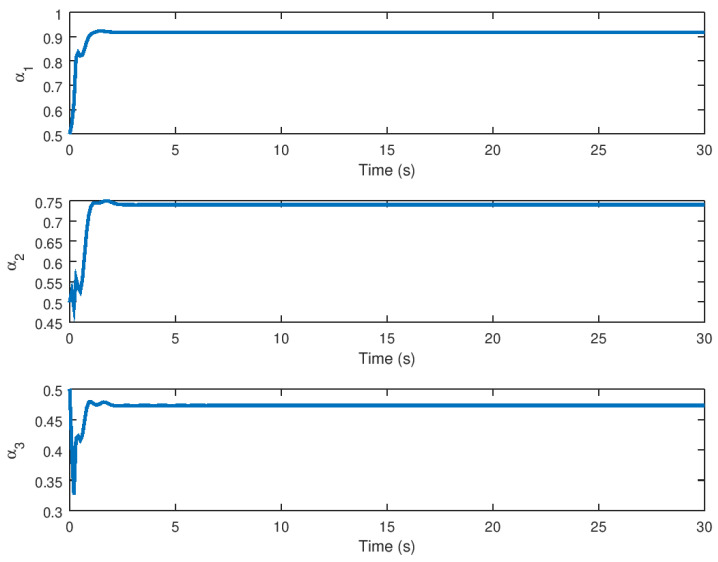
Evolution in time of the α variable for synchronization purposes.

Finally, in Figure 15 the evolution in time of the variable α for stabilization purposes is depicted, with the variable reaching equilibrium when synchronization is completed. This variable, like the extra state variable in the control technique for stabilization purposes, is basically the extra state variable with input *v*, as shown in Figure 14. Note that this auxiliary variable aids in the stability of the novel chaotic system in the presence of input saturation nonlinearity, which is significant given that this variable is appropriate for implementation in real physical systems in order to synchronize two identical systems, which in this case are essentially the novel chaotic system presented in this paper.

### 5.3. Experimental Results Analysis

The experimental results for stabilization and control purposes, as explained in this section, numerically corroborate the results obtained in this study. It is possible to see how the variables reach equilibrium while suppressing chaotic behavior in the stabilization experiment. The three variables *x*, *y*, and *z* of the stabilized chaotic system are shown in Figure 5 how these variables reach the equilibrium point in a fast and accurate way in approximately 1 *s* despite the input saturation that is found in the chaotic system input providing a stable response in finite time. This is an important consideration, especially if this controller is used in a real-world physical system. It is demonstrated in Figure 1 how the proposed controller drives the state variable to a stable equilibrium rather than, as shown in Figure 1, to a chaotic region of attraction suppressing the limit cycle. The time evolution of the input variable *U*, as shown in Figure 7 depicts how a small control effort is generated in order to avoid input saturation of the stabilized chaotic system, which is important because input saturation suppresses some phenomena such as instability or chattering.

Meanwhile, the evolution in time of the extra additional input variable *v* and the state variable α can be seen in Figure 8 and Figure 9 demonstrating that these variables reach their respective equilibrium until the chaotic system variables are stabilized. In the case of synchronization, it can be seen how the response system follows the drive variable in finite time, reducing the error to zero, as shown in Figure 10 and Figure 11, respectively, given that it is difficult to drive the synchronization error variable in the presence of saturation while avoiding chattering or instability. It is also important to note in Figure 13 that the synchronization input variable provides an anti chattering response and a small control effort in a very short time until the proposed control synchronization law achieves ideal synchronization. Finally, as in the stabilization case, the variables *v* and α, as shown in Figure 14 and Figure 15 depict the time evolution of the auxiliary variables, indicating how these variables reach the equilibrium.

## 6. Discussion

Taking into account the theoretical results, note that a novel chaotic system is created by combining two sigmoidal functions. It can be seen that a chaotic attractor is designed in which, as confirmed, the domain of attraction of this chaotic system is the equilibrium point, resulting in this chaotic system being a self-excited chaotic system. The proposed novel chaotic system is well suited to modeling many chaotic behaviors observed in nature and physical systems. The bifurcation diagrams demonstrate the chaotic behavior of this system as it transitions from a stable to a chaotic regime, and vice versa.

The robust controller is implemented appropriately for stabilization purposes by selecting an appropriate Lyapunov functional. The addition of an additional system provides the required inputs, which are considered dynamic inputs and include the discontinuity as part of the overall system input with saturation. The saturation is modeled by implementing the sector condition while accounting for the deadzone nonlinearity and taking into account the dynamic characteristics of this input nonlinearity. The conditions for obtaining an appropriate control law are provided by an anti-chattering stabilization control law and a state feedback input. A similar axiomatic methodology is used to find the robust control synchronization law by first defining the errors in the system dynamics and then selecting the appropriate robust control Lyapunov function.

In the case of the novel chaotic system’s robust stabilization, the state feedback controller part moves the eigenvalues of the linearized novel chaotic system to a point where the state variables reach equilibrium in finite time, while the robust controller part switches this input variable to obtain a fast, accurate, and reliable response. Something similar happens with this novel chaotic system’s robust control synchronization law. The linearized error system’s eigenvalues are moved to an appropriate position in order to drive this error variable to zero in finite time. A state feedback controller is implemented to reach the zero value of the state variable while avoiding the input system chattering.

Meanwhile, in the experimental results section, it is possible to see how the robust controller drives the state variables of the stabilized system to the equilibrium point in finite time, with small peaks, and without chattering. When input saturation is detected in the system input, the small control effort generated by the variables *U* and *v* is crucial. Despite the fact that the input saturates at very low values, the system variables reach the equilibrium point in finite time, avoiding instability and, more importantly, poor performance. As seen in the synchronization case of this drive and response novel chaotic system, the drive and response variables are synchronized more quickly, accurately reaching the zero error between these variables. In the case of synchronization, saturation has no effect on synchronization performance, avoiding instability or poor performance, which is desirable given that saturation is almost unavoidable in most physical systems.

Given the vast number of physical systems in which the chaos phenomenon can be found, the results presented in this study can be implemented without difficulty in any of these physical systems represented or modeled as dynamic systems. One of the main advantages is that the chaos suppression robust control strategy for stabilization effectively eliminates nonlinear saturation effects on this novel chaotic system, such as instability of degraded performance, while also efficiently stabilizing the novel chaotic system’s equilibrium points.

Apart from whether this control strategy is implemented in any type of physical system, the results provided in this research study can be extended to various types of physical systems in which the chaotic phenomenon is found, such as the physical systems mentioned previously in this research study. These results can be modified so that the robust controller can be implemented in other systems where hyperchaos or other phenomena are present. Note that the results obtained in this study can be extended for chaos synchronization in various types of systems in which not only two systems are synchronized, because it can be extended to chaos synchronization for multi-coupled chaotic systems. Furthermore, the results obtained in this study can be easily extended to complex chaotic networks, where it is important to note that robust control is always necessary given the vast number of physical systems in which chaotic synchronization is required.

## 7. Conclusions

This paper presents a novel chaotic attractor, as well as its robust control stabilization and synchronization with input saturation. First, the novel attractor is presented, in which two sigmoidal functions are implemented to generate chaotic dynamics, followed by a bifurcation analysis in which the stability and chaotic regimen regions for the novel chaotic attractor’s parameters *a*, *b*, and *d* are presented. The phase portraits plot confirms that this chaotic attractor reaches the domain of attraction at the equilibrium point, despite the fact that it is self-excited. The control laws are obtained by using an extra system after a robust controller for stabilization purposes is derived by considering the sector condition property of the saturation input and implementing a robust control Lyapunov function. To obtain the synchronization control law, a similar methodology is used, but this time the synchronization error variable is established. Finally, two numerical experiments are carried out in conjunction with the research study’s respective discussions and conclusions. The results presented in this numerical experiment section demonstrate that, in the stabilization case, the novel chaotic system can be driven to the equilibrium points in finite time with very little control effort and no oscillation or chattering. Note that the resulting control stabilization law can be efficiently implemented even in many different types of physical systems, such as electrical mechanical or other systems found in exact sciences. As in the stabilization case, it is demonstrated that in the synchronization case, an efficient robust control synchronization law is used, even in the presence of an input saturation nonlinearity in the response system, and that synchronization between the drive and response systems is achieved faster and more accurately. A gain matrix will be included in the stabilization and control laws in the future to be tuned by linear matrix inequalities.

## Data Availability

Not applicable.
